# Relaxation Time Mapping of Early‐Stage Osteonecrosis of the Femoral Head: An In Vivo Piglet Model Study

**DOI:** 10.1002/jor.70236

**Published:** 2026-06-04

**Authors:** Casey P. Johnson, Erick O. Buko, Sampada Bhave, Kayla L. Chase, Douglas Albrecht, Alexandra R. Armstrong, Jutta M. Ellermann, Harry K. W. Kim, Ferenc Tóth

**Affiliations:** ^1^ Department of Veterinary Clinical Sciences University of Minnesota St. Paul Minnesota USA; ^2^ Center for Magnetic Resonance Research University of Minnesota Minneapolis Minnesota USA; ^3^ Department of Radiology University of Minnesota Minneapolis Minnesota USA; ^4^ Center of Excellence in Hip Scottish Rite for Children Dallas Texas USA; ^5^ Department of Orthopedic Surgery UT Southwestern Medical Center Dallas Texas USA

**Keywords:** ischemia, Legg–Calvé–Perthes disease, osteonecrosis, quantitative magnetic resonance imaging, relaxation time mapping

## Abstract

Quantitative magnetic resonance imaging (MRI) relaxation time mapping techniques may be useful to evaluate early‐stage osteonecrosis of the femoral head, particularly in children with Legg–Calvé–Perthes disease (LCPD). The purpose of this study was to assess the sensitivities of T2, T1ρ, adiabatic T1ρ (aT1ρ), and adiabatic T2ρ (aT2ρ) relaxation time mapping to detect early‐stage ischemic injury to the secondary ossification center (SOC) of the femoral head in vivo in a piglet model of LCPD. We hypothesized that relaxation times would be increased in the SOCs of ischemic versus contralateral‐control femoral heads 1 week following onset of ischemia. Bilateral hips of *n* = 13 piglets with confirmed, surgically‐induced complete ischemia of the SOC were imaged 1 week post‐operatively using a clinical 3 T MRI scanner. Median T2 (*n* = 13), T1ρ (*n* = 13), aT1ρ (*n* = 10), and aT2ρ (*n* = 10) relaxation times were measured for the total SOC, as well as its central and peripheral subregions, and were compared between the ischemic versus control femoral heads using paired *t*‐tests (*p* < 0.05). All four relaxation times were significantly increased in the ischemic versus control femoral heads, with a more pronounced increase in the peripheral versus central subregion of the SOC. T2 and aT2ρ had the greatest percent increases and effect sizes of the four relaxation times. In conclusion, T2, T1ρ, aT1ρ, and aT2ρ relaxation time mapping techniques are sensitive in detecting ischemic injury to bone marrow of the femoral head in vivo in a piglet model of LCPD at clinical 3 T MRI field strength.

## Graphical abstract

T2, T1ρ, adiabatic T1ρ, and adiabatic T2ρ relaxation time mapping techniques are sensitive in detecting ischemic injury to bone marrow of the femoral head in vivo in a piglet model at 3 T MRI. T2 and adiabatic T2ρ relaxation times have the most pronounced increase in ischemic versus perfused, contralateral‐control femoral heads 1 week after induction of ischemia. These quantitative MRI techniques may aid in quantitative evaluation and monitoring of early‐stage ischemic injury in patients with osteonecrosis of the femoral head.

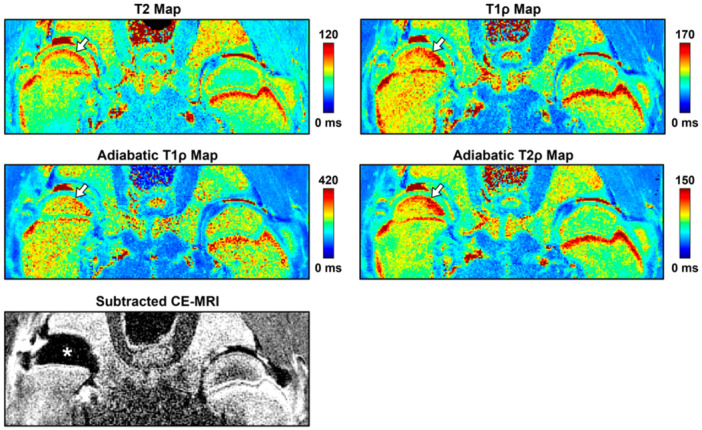

## 1 Introduction

Osteonecrosis of the femoral head (ONFH) is a major cause of hip osteoarthritis and is characterized by ischemic injury to the bony epiphysis that can lead to its mechanical weakening and collapse [1, 2]. ONFH afflicts both children and adults; the juvenile, idiopathic form of ONFH is called Legg–Calvé–Perthes disease (LCPD), and it is one of the most common hip disorders in children that produces a permanent femoral head deformity [3, 4]. Treatment outcomes for patients with ONFH are improved when implemented early in the disease process (e.g., during the early, avascular phase, prior to femoral head collapse) [2, 5, 6], thus early detection and characterization of the severity of ischemic injury to the femoral head are of critical importance. However, current clinical imaging methods (radiographs and conventional T1‐ and T2‐weighted MRI) have limited ability to detect and characterize the severity of ONFH in its earliest stages [7, 8]. In adults, MRI sensitivity and specificity for ONFH approach 100% once the characteristic band or double‐line sign appears, but diagnostic accuracy is substantially lower in the earliest stage, when marrow changes are absent or non‐specific and cannot be reliably distinguished from transient bone marrow edema or subchondral insufficiency fracture [7, 9]. Additionally, inter‐observer agreement for MRI‐based staging of adult ONFH is moderate to substantial at best (with kappa values ranging from 0.57 to 0.72) [10, 11], reflecting the inherently qualitative nature of current severity metrics. In children with ONFH, the double‐line sign is not seen; thus, contrast‐enhanced MRI (CE‐MRI) is used to detect femoral head hypoperfusion and provide an early quantitative measure of disease severity [8, 12, 13, 14].

Quantitative MRI techniques, which measure tissue properties rather than providing qualitative contrast, may be advantageous for earlier, more specific, complementary, and/or longitudinal evaluation of ONFH in both children and adults compared to conventional MRI. Several quantitative MRI techniques have been proposed to assess early‐stage ONFH, including dynamic CE‐MRI and quantitative mapping of apparent diffusion coefficient using diffusion‐weighted imaging [12, 15, 16, 17, 18, 19, 20, 21, 22]. In LCPD, while CE‐MRI has become a routine clinical approach to quantitatively evaluate the extent of femoral head hypoperfusion [8, 13], concerns about the unknown effects of gadolinium deposition in the brain limit its repeated use in children [23]. Furthermore, while CE‐MRI can assess femoral head perfusion, changes in the blood flow to the femoral head are not necessarily correlated with the severity of injury to the bone marrow and bone, which may also be useful, complementary information to predict clinical outcome and inform treatment decisions. Diffusion‐weighted imaging is a non‐contrast‐enhanced technique that can be used to evaluate ischemic injury to the femoral head, but it has relatively low spatial resolution, further highlighting the need for alternative approaches.

Quantitative mapping of T2 and T1ρ relaxation times is a potential alternative, non‐contrast‐enhanced MRI solution to image ischemic injury to the femoral head. T2 and T1ρ relaxation time mapping have been shown, in ex vivo studies of a piglet model of LCPD conducted at 9.4 T MRI, to be sensitive in detecting injury to the femoral head as early as 48 h after onset of ischemia [24, 25]. T1ρ mapping was particularly sensitive in detecting ischemic injury to the secondary ossification center (SOC; i.e., the bone and bone marrow of the developing femoral epiphysis) in these ex vivo studies. This was attributed to greater sensitivity of T1ρ mapping to slow molecular processes that may be impacted by acidosis, increased intracellular water, and/or rupture of cell membranes, suggesting that T2 and T1ρ mapping may serve as complementary techniques to provide more specific information about changes within the injured SOC [24, 25]. T2 and T1ρ mapping have also been shown in LCPD piglet model studies to be sensitive in detecting injury to the epiphyseal cartilage overlying the SOC as well as to the metaphyseal spongiosa distal to the growth plate [26, 27]. Thus, T2 and T1ρ mapping provide a potential means for relatively highly‐resolved, comprehensive assessment of ischemic injury to the femoral head. Adiabatic T1ρ (aT1ρ) and adiabatic T2ρ (aT2ρ) relaxation time mapping techniques may also be advantageous for *in vivo* imaging of the femoral head, given their unique contrast mechanisms and ability to image with reduced radiofrequency power demands compared to continuous‐wave T1ρ methods [28, 29, 30]. aT1ρ samples a broader range of spin‐lock frequencies than continuous‐wave T1ρ and thus may probe unique changes in slow molecular processes, while aT2ρ provides increased sensitivity to the diffusion of water across susceptibility gradients, such as those of trabecular bone, which may improve its sensitivity to changes within the ischemic femoral head [28, 29, 31].

The purpose of this study was to assess the sensitivities of T2, T1ρ, aT1ρ, and aT2ρ relaxation time mappings to detect early‐stage ischemic injury to the SOC in a piglet model of LCPD in vivo using a clinical 3 T MRI scanner. We evaluated the relaxation times in the SOC as a whole as well as in central and peripheral subregions comprised of relatively mature and immature bone and bone marrow, respectively. We hypothesized that the four relaxation times would be increased in the SOCs of the ischemic versus contralateral‐control femoral heads 1 week following the onset of ischemia.

Data from this same cohort of piglets have been previously reported for intravoxel incoherent motion (IVIM) diffusion‐weighted imaging in the SOC, as well as relaxation time mapping of the epiphyseal cartilage and metaphyseal spongiosa [26, 27, 32, 33]. This study uniquely focuses on relaxation time mapping of the SOC.

## 2 Methods

### 2.1 Animal Model

Our study was approved by the University of Minnesota's Institutional Animal Care and Use Committee. Fourteen Yorkshire piglets (eight male and six female) were obtained from a commercial provider (Manthei Hog Farm, LLC; Elk River, MN). Piglets were pair‐housed in pens and fed a standard diet at the University of Minnesota's Research Animal Resources facilities. At 6 weeks of age (mean weight = 10.2 ± 1.8 kg; weight range = 8.1–14.5 kg), the piglets underwent surgery to induce complete ischemia of the SOC of the femoral head by placing a ligature around the femoral neck and transecting the ligamentum teres [34, 35]. The contralateral femoral head was unoperated and served as a control. The piglets were imaged at 3 T MRI 1 week after surgery. For both the surgical and MRI procedures, the piglets were sedated using intramuscular injection of either midazolam (10 mg/kg) and buprenorphine (10 μg/kg) or telazol (4.0 mg/kg) and xylazine (2.0 mg/kg), then anesthetized using intravenous administration of ketamine (5.0 mg/kg) or propofol (2.0–6.0 mg/kg). General anesthesia was maintained by inhalation of isoflurane (1.0%–5.0%) vaporized in oxygen. Post‐operative care included oral administration of carprofen (2.0–3.0 mg/kg) once or twice daily for 3 days for pain control. Five of the first six pigs studied (Pigs 1, 2, 3, 5, and 6) were slated (prior to the experimental procedures) for histological analyses of the femoral heads and were euthanized immediately following the MRI exam with intravenous administration of potassium chloride (75–150 mg/kg) or sodium pentobarbital (100 mg/kg), while the other nine piglets (Pigs 4 and 7–14) were recovered for a different study.

### 2.2 In Vivo 3 T MRI

The piglets were imaged in vivo under general anesthesia using a 3 T MRI system (MAGNETOM Prisma; Siemens Healthcare; Erlangen, Germany) and a pair of vendor‐provided four‐channel small flex receiver coil arrays. The operated and control femoral heads were imaged simultaneously with the sequences and parameters listed in Table 1. First, 2D T2 and T1ρ relaxation time maps were acquired for all *n* = 14 piglets, and 2D aT1ρ and aT2ρ maps were acquired for the latter *n* = 11 piglets using a magnetization‐prepared 2D turbo spin‐echo (TSE) sequence. The 2D relaxation time maps were acquired at a single slice passing through the centers of both femoral heads to provide both relatively high signal‐to‐noise ratio and in‐plane spatial resolution. Second, 3D T2 and T1ρ maps were acquired for all *n* = 14 piglets using a magnetization‐prepared SPACE (“sampling perfection with application optimized contrasts using different flip angle evolution”) sequence to test whether differences in the ischemic versus control femoral heads were similar across the entire femoral head as compared to the higher‐resolution, single‐slice 2D maps. The 3D mapping sequence was modified for Pigs 9–14 versus Pigs 1–8, resulting in different magnetization preparation times, but this is not expected to have a significant effect on the difference measurements [36]. Third, a high‐resolution, morphological 3D GRE sequence was acquired for 3D segmentation of the SOC. Lastly, to confirm surgical induction of complete (100%) ischemia of the SOC, subtraction CE‐MRI was acquired using a 2D multi‐slice T1‐weighted TSE sequence repeated before and 1 min after intravenous administration of 0.2 mmol/kg gadoteridol (ProHance; Bracco Diagnostics; Monroe Township, NJ).

**Table 1:** 3 T MRI imaging parameters.

	2D Mapping: T2 and T1ρ (Pigs 1–14)	2D Mapping: aT1ρ and aT2ρ (Pigs 4–14)	3D Mapping: T2 and T1ρ (Pigs 1–8)	3D Mapping: T2 and T1ρ (Pigs 9–14)	3D SOC segmentation^a^	CE‐MRI
Sequence	Magnetization‐prepared 2D TSE	Magnetization‐prepared 2D TSE	Magnetization‐prepared 3D SPACE	Magnetization‐prepared 3D SPACE	3D GRE	2D TSE
Field‐of‐view (mm)	200 × 200	200 × 200	200 × 200 × 128	200 × 150 × 40	200 × 150 × 44	200 × 170
Sampling matrix	384 × 384	384 × 384	384 × 384 × 128	384 × 288 × 40	512 × 384 × 112	448 × 380
Resolution (mm)	0.52 × 0.52	0.52 × 0.52	0.52 × 0.52 × 1.0	0.52 × 0.52 × 1.0	0.39 × 0.39 × 0.39	0.45 × 0.45
Slices/Thickness (mm)	1/2.0	1/2.0				20/2.0
TR/TE (ms)	2230/13	2230/13	2610/163	2500/217	31.0/22.5	970/21
Flip angle (degrees)	180	180	Variable	Variable	15	150
Bandwidth (Hz/px)	130	130	501	501	100	260
Fat sat	No	No	No	No	No	Yes
GRAPPA			*R* = 2	*R* = 2		*R* = 2
Turbo factor	8	8	128	128		4
Preparation pulses	MLEV4; 350 Hz spin‐lock	HS1, 6 ms, 1000 Hz max	MLEV4; 350 Hz spin‐lock	MLEV4; 300 Hz spin‐lock		
Prep times (ms)	0, 20, 40, 60, 80	0, 24, 48, 72, 96	0, 80	0, 20, 40, 60		
*M*_z_ recovery time (ms)	2000	2000	2000	1850		
Scan time	9:24 (per map)	9:24 (per map)	14:21 (per map)	16:40 (per map)	17:26	3:10 (×2 images)

Abbreviations: aT1ρ, adiabatic T1ρ; aT2ρ, adiabatic T2ρ; CE‐MRI, contrast‐enhanced MRI; GRAPPA, generalized autocalibrating partially parallel acquisitions; GRE, gradient echo; HS1, hyperbolic secant pulse; MLEV4, Malcolm–Levitt phase cycling pulse; MRI, magnetic resonance imaging; SOC, secondary ossification center; SPACE, sampling perfection with application optimized contrasts using different flip angle evolution; TE, echo time; TR, repetition time; TSE, turbo spin echo

^a^ The 3D GRE sequence parameters varied slightly between pigs, but were all acquired with the same spatial resolution. Shown are typical parameters.

### 2.3 Histology

Femoral heads were harvested from the subset of *n* = 5 euthanized piglets for histological analysis. Each specimen was bisected in the coronal plane with a diamond bone saw using the apex of the greater trochanter and the fovea as landmarks for consistent sectioning. Specimens were then fixed in 10% neutral buffered formalin and decalcified in 10% ethylenediaminetetraacetic acid. A 2‐mm‐thick mid‐coronal slab was then cut and embedded in paraffin. A single section, which included the entire extent of the femoral head in the mid‐coronal plane, was stained with hematoxylin and eosin (H&E). Each histological section was qualitatively assessed by a board‐certified veterinary pathologist with expertise in comparative orthopedics (A.R.A.), who was blinded to surgery. The entire extent of the SOC was examined for each operated and control femoral head, and any pathological changes, as well as any regional differences in the central versus peripheral subregions of the SOC, were noted.

### 2.4 Data and Statistical Analysis

**Figure 1:**
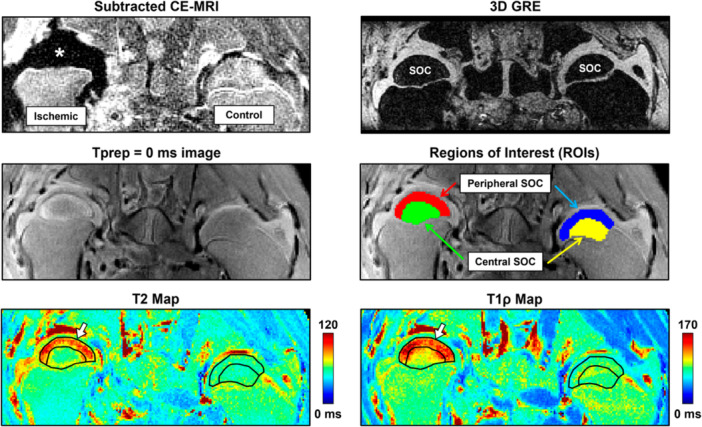
Overview of the MRI analysis procedure. Subtraction contrast‐enhanced MRI (CE‐MRI) was used to confirm ischemia (i.e., lack of contrast uptake) in the secondary ossification center (SOC; i.e., the epiphyseal bone and bone marrow) of the operated femoral head (asterisk). 3D GRE images and weighted images from the 2D T2 mapping acquisitions were used to segment the SOC for the ischemic and control femoral heads. The total SOC region was then divided into central (inner 2/3) and peripheral (outer 1/3) subregions. Median relaxation times were then calculated in the total SOC and central and peripheral subregions. The ROI boundaries are overlaid on 2D T2 and T1ρ maps for one of the piglets. In this case, relaxation times can be seen to be increased in the SOC of the ischemic versus contralateral‐control femoral head, particularly in the peripheral subregion (white arrows).

2D and 3D relaxation time maps were generated by fitting a two‐parameter mono‐exponential signal decay model using MATLAB (2025a; MathWorks; Natick, Massachusetts). 2D and 3D regions of interest (ROIs) were manually segmented for the SOCs of the ischemic and control femoral heads using ITK‐SNAP (www.itksnap.org) [37]. First, the total SOC was segmented, and then it was divided into two subregions: (i) the central portion, defined as the inner 2/3 of the SOC radius; and (ii) the peripheral portion, defined as the outer 1/3 of the SOC radius (Figure 1). The 2D segmentations were defined using the *T*_prep_ = 0 ms images from the T2 mapping acquisitions. The 3D segmentations were defined using the 3D GRE images, and either 3D Slicer (www.slicer.org) [38] or MATLAB was used to register the 3D relaxation time maps to the 3D GRE images using DICOM header spatial information and linear interpolation. Each registered 3D quantitative map was visually verified to have accurate alignment with the reference 3D GRE images and corresponding segmentations. Median relaxation time values for the 2D and 3D relaxation time maps were measured within each ROI using MATLAB, and they were then statistically compared between the pairs of ischemic and contralateral‐control femoral heads using two‐tailed paired *t*‐tests (*p* < 0.05 with Holm–Bonferroni correction for multiple comparisons) with R statistical software (Version 4.3.1) [39]. Mean percent differences between the median values of the paired ischemic and control femoral heads and effect sizes (Cohen's *d*) were calculated and compared between the four different relaxation times to assess their relative responses to injury in the ischemic femoral heads.

## 3 Results

Thirteen of the 14 of the piglets had complete femoral head ischemia as confirmed by CE‐MRI and were included in the analysis. One piglet (Pig 10, a male) had only partial femoral head ischemia and was excluded. This left *n* = 13 piglets (seven male and six female) that were imaged with 2D and 3D T2 and T1ρ mapping, *n* = 10 of which (Pigs 4–14, excluding Pig 10) were additionally imaged with 2D aT1ρ and aT2ρ mapping, and *n* = 5 of which (Pigs 1, 2, 3, 5, and 6) were assessed with histology.

**Figure 2:**
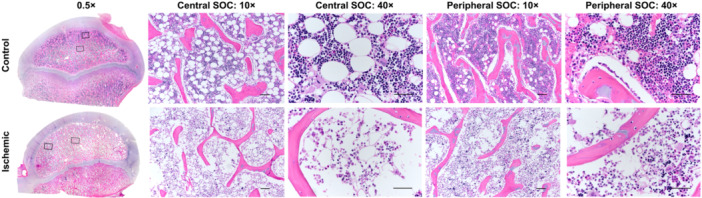
Photomicrographs of hematoxylin and eosin (H&E)‐stained sections of the epiphyseal bone and marrow of a representative pair of control and ischemic femoral heads. Photomicrographs are shown at magnifications of 0.5×, 10×, and 40× for the central and peripheral subregions of the secondary ossification centers (SOCs). The ischemic femoral head had widespread necrosis of bone marrow cells and more limited and regionally variable necrosis of osteocytes. The central and peripheral subregions of the SOC in the ischemic femoral head had a similar extent of necrosis, despite the central subregion in the control sample having more mature bone and bone marrow than the peripheral subregion, including a greater number of adipocytes and reduced cellularity of erythroid and myeloid precursor lineage cells and megakaryocytes. The boxes on the 0.5× magnified images indicate the locations of the 10× magnified images of the central (dashed boxes) and peripheral (solid boxes) subregions. Scale bars indicate 100 and 50 µm for the 10× and 40× magnified images, respectively.

On histology, the five ischemic femoral heads analyzed each had extensive necrosis of bone marrow cells and incomplete, regionally variable necrosis of osteocytes (osteonecrosis). The extent and morphology of injury in each of these ischemic femoral heads appeared similar in the central and peripheral subregions of the SOC, regardless of the variation in cellular composition of these subregions. In the five paired control samples, no pathologic changes were noted, but the peripheral subregion of the SOC had less adipose tissue and more myeloid and erythroid precursors, bone marrow stromal cells, red blood cells, and smaller caliber vessels than the more mature, central subregion. There were no notable differences in the histological findings between different animals. H&E‐stained photomicrographs for the ischemic and control femoral heads of one of the piglets are shown in Figure 2, the results of which are representative of the findings for each of the animals.

**Figure 3:**
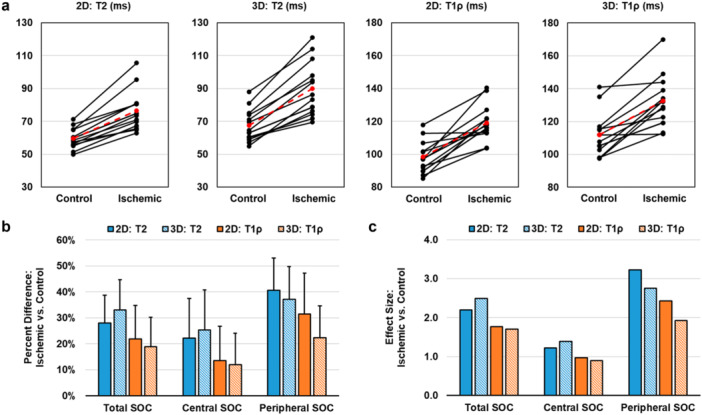
Differences in T2 and T1ρ relaxation times between *n* = 13 pairs of ischemic and control femoral heads in the secondary ossification center (SOC) and its central and peripheral subregions using 2D and 3D quantitative mapping sequences. (a) Paired differences in the relaxation times in the total SOC using 2D and 3D mapping sequences. Pairs of data for individual piglets are indicated by black lines, and average differences are indicated by dashed red lines. (b) Percent differences in the relaxation times between the ischemic and control femoral heads. The percent differences were greater for T2 than T1ρ in the total SOC and its central and peripheral subregions. Similar results were seen using the 2D and 3D sequences, supporting that the effects are consistent across the full volume of the SOC. Error bars indicate standard deviation. (c) Effect sizes of the paired differences. The effect sizes were greater for the T2 versus T1ρ relaxation times, and they were also similar between the 2D and 3D sequences.

In the *n* = 13 piglets assessed with T2 and T1ρ mapping, both relaxation times were significantly increased in the ischemic versus control femoral heads (Table 2 and Figure 3). Differences in relaxation times between the ischemic and control femoral heads were similar using the 2D and 3D mapping acquisitions. For both sequences, T2 and T1ρ were significantly increased in the total SOC (*p* < 0.0001) as well as its peripheral (*p* < 0.0001) and central (*p* ≤ 0.0072) subregions. The increase in relaxation times was more pronounced in the peripheral than the central subregion of the SOC. In all ROIs, T2 had a greater percentage increase and effect size than T1ρ. Comparing the T2 and T1ρ difference measurements between the 2D and 3D mapping acquisitions in each of the three ROIs using paired *t*‐tests (*p* < 0.05, uncorrected), only the change in T2 in the total SOC was statistically different (5.6 ± 6.7 ms greater using the 3D vs. 2D sequence; *p* = 0.011).

**Table 2:** Paired differences in T2 and T1ρ relaxation times between *n* = 13 pairs of ischemic and control femoral heads 1 week after onset of ischemia.

Region of interest (ROI)	Relaxation time	Control (ms)	Ischemic (ms)	Paired difference (ms)	95% CI (ms)	*t*	*p* value	Effect size (Cohen's *d*)	Percent increase
2D TSE sequence									
Total SOC	T2	59.5 ± 6.4	76.4 ± 12.5	16.9 ± 7.7	[12.3, 21.6]	7.9	< 0.0001*	2.2	28 ± 11%
	T1ρ	98.4 ± 9.7	119.2 ± 11.2	20.8 ± 11.8	[13.7, 27.9]	6.4	< 0.0001*	1.8	22 ± 13%
Central SOC	T2	56.4 ± 6.6	69.4 ± 15.5	13.0 ± 10.6	[6.6, 19.4]	4.4	0.0008*	1.2	22 ± 15%
	T1ρ	98.4 ± 11.3	111.0 ± 12.9	12.5 ± 12.9	[4.7, 20.3]	3.5	0.0044*	1.0	13 ± 13%
Peripheral SOC	T2	62.4 ± 6.9	87.5 ± 10.9	25.1 ± 7.8	[20.4, 29.8]	11.6	< 0.0001*	3.2	41 ± 12%
	T1ρ	98.0 ± 9.7	127.9 ± 10.3	29.8 ± 12.3	[22.4, 37.3]	8.7	< 0.0001*	2.4	31 ± 16%
3D SPACE sequence									
Total SOC	T2	67.5 ± 9.9	90 ± 16.7	22.5 ± 9.0	[17.0, 27.9]	9.0	< 0.0001*	2.5	33 ± 12%
	T1ρ	112.1 ± 13.4	132.7 ± 15.7	20.6 ± 12.0	[13.3, 27.9]	6.2	< 0.0001*	1.7	19 ± 11%
Central SOC	T2	60.8 ± 12.1	76.5 ± 19.4	15.7 ± 11.3	[8.8, 22.5]	5.0	0.0003*	1.4	25 ± 15%
	T1ρ	111.7 ± 15.1	124.2 ± 15.4	12.5 ± 13.9	[4.1, 20.9]	3.2	0.0072*	0.9	12 ± 12%
Peripheral SOC	T2	70.3 ± 9.2	96.5 ± 16.3	26.2 ± 9.5	[20.4, 31.9]	9.9	< 0.0001*	2.8	37 ± 13%
	T1ρ	112.5 ± 13.2	137.1 ± 16.7	24.5 ± 12.8	[16.8, 32.3]	6.9	< 0.0001*	1.9	22 ± 12%

*Note:* Values are reported as mean ± standard deviation.

Abbreviation: SOC, secondary ossification center.

* Statistically significant after Holm–Bonferroni correction for six comparisons.

**Figure 4:**
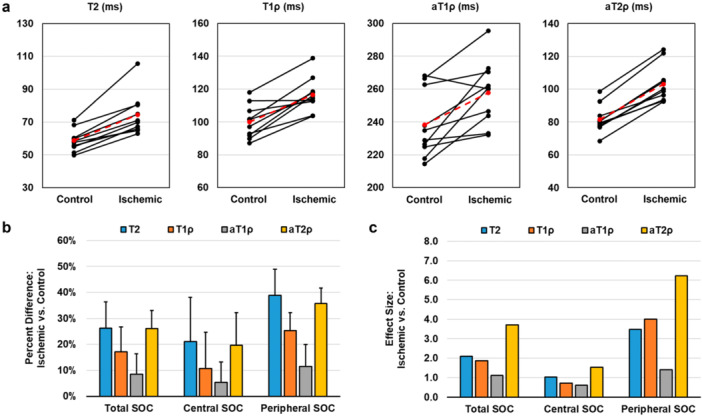
Differences in T2, T1ρ, adiabatic T1ρ (aT1ρ), and adiabatic T2ρ (aT2ρ) relaxation times between *n* = 10 pairs of ischemic and control femoral heads in the secondary ossification center (SOC) and its central and peripheral subregions using 2D quantitative mapping sequences. (a) Paired differences in the four relaxation times in the total SOC. Pairs of data for individual piglets are indicated by black lines, and average differences are indicated by dashed red lines. (b) Percent differences in the relaxation times between the ischemic and control femoral heads. T2 and adiabatic T2ρ had greater relative increases than T1ρ and aT1ρ in all three regions of interest. For all four relaxation times, the increases were most pronounced in the peripheral subregion of the SOC. Error bars indicate standard deviation. (c) Effect sizes of the paired differences. aT2ρ had the greatest effect size of all of the relaxation times in each region of interest.

In the subset of *n* = 10 piglets additionally assessed with aT1ρ and aT2ρ, all four relaxation times significantly increased in the ischemic versus control femoral heads (Table 3 and Figure 4). Each relaxation time was significantly increased in the total SOC (*p* ≤ 0.0069) and its central (*p* ≤ 0.0099) and peripheral (*p* ≤ 0.0016) subregions, with the exception of T1ρ and aT1ρ in the central subregion (*p* = 0.049 and 0.089, respectively). Of the four relaxation times, T2 and aT2ρ had the greatest percent increases, while aT2ρ had the largest effect sizes, in all ROIs in response to ischemic injury. aT1ρ had the most variable responses to ischemic injury to the SOC and the lowest percent increases and effect sizes of the four relaxation times.

**Table 3:** Paired differences in T2, T1ρ, adiabatic T1ρ (aT1ρ), and adiabatic T2ρ (aT2ρ) relaxation times between *n* = 10 pairs of ischemic and control femoral heads 1 week after the induction of ischemia.

Region of interest (ROI)	Relaxation time	Control (ms)	Ischemic (ms)	Paired difference (ms)	95% CI (ms)	*t*	*p* value	Effect size (Cohen's *d*)	Percent increase
Total SOC	T2	58.8 ± 6.7	74.4 ± 12.6	15.6 ± 7.5	[10.3, 21.0]	6.6	0.0001*	2.1	26 ± 10%
	T1ρ	100 ± 10	117 ± 10	16.6 ± 9.0	[10.2, 23.0]	5.9	0.0002*	1.9	17 ± 10%
	aT1ρ	238 ± 20	258 ± 20	20 ± 18	[6.8, 32.1]	3.5	0.0069*	1.1	8.5 ± 7.8%
	aT2ρ	81.7 ± 8.4	103 ± 12	21.3 ± 5.7	[17.2, 25.4]	11.8	< 0.0001*	3.7	26.1 ± 6.9%
Central SOC	T2	55.2 ± 6.9	67 ± 17	12 ± 12	[3.7, 20.4]	3.2	0.0099*	1.0	21 ± 17%
	T1ρ	99 ± 12	109 ± 13	9.7 ± 13.5	[0.0, 19.4]	2.3	0.049	0.7	11 ± 14%
	aT1ρ	238 ± 22	250 ± 20	12 ± 19	[−2.2, 25.4]	1.9	0.089	0.6	5.2 ± 8.0%
	aT2ρ	80.3 ± 8.8	96 ± 14	16 ± 10	[8.3, 22.7]	4.9	0.0009*	1.5	20 ± 13%
Peripheral SOC	T2	62.1 ± 7.3	86 ± 11	24.0 ± 6.9	[19.1, 28.9]	11.0	< 0.0001*	3.5	39 ± 10%
	T1ρ	100.5 ± 8.6	125.7 ± 9.7	25.2 ± 6.3	[20.7, 29.7]	12.7	< 0.0001*	4.0	25.3 ± 6.8%
	aT1ρ	238 ± 20	265 ± 22	26 ± 19	[13.0, 39.9]	4.4	0.0016*	1.4	11.4 ± 8.6%
	aT2ρ	83.2 ± 8.1	113 ± 10	29.5 ± 4.7	[26.0, 32.8]	19.6	< 0.0001*	6.2	35.6 ± 6.0%

*Note:* Values are reported as mean ± standard deviation.

Abbreviation: SOC, secondary ossification center.

* Statistically significant after Holm–Bonferroni correction for 12 comparisons.

**Figure 5:**
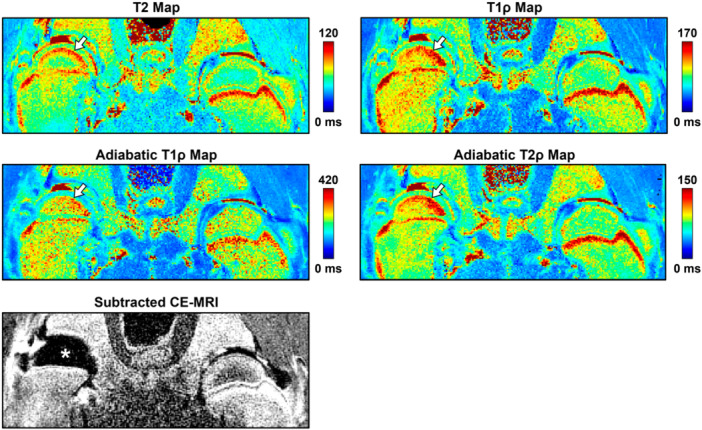
2D relaxation time maps and subtracted contrast‐enhanced MRI (CE‐MRI) image for a piglet imaged one week after the onset of femoral head ischemia. The ischemic femoral head and its secondary ossification center (SOC) are indicated by the arrows and asterisk. T2, T1ρ, adiabatic T1ρ, and adiabatic T2ρ relaxation times were all increased in the ischemic versus perfused contralateral‐control femoral head. There was a more pronounced increase in relaxation times in the peripheral versus the central subregion of the SOC.

**Figure 6:**
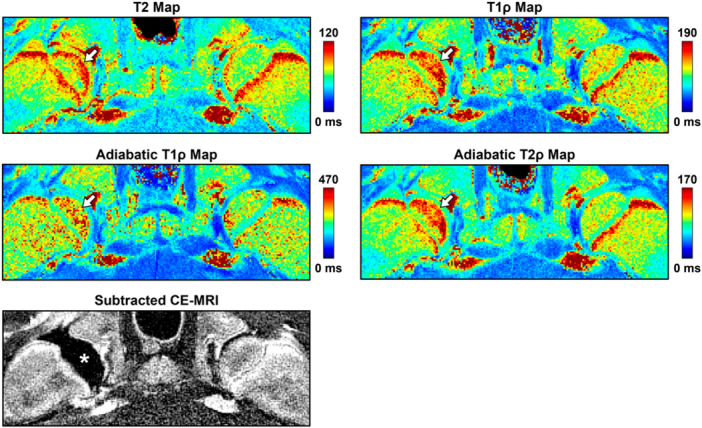
2D relaxation time maps and subtracted contrast‐enhanced MRI (CE‐MRI) image for a second piglet imaged one week after the onset of femoral head ischemia. The images are displayed as in Figure 5. This case also shows a more pronounced increase in the relaxation times in the peripheral versus the central subregion of the secondary ossification center.

**Figure 7:**
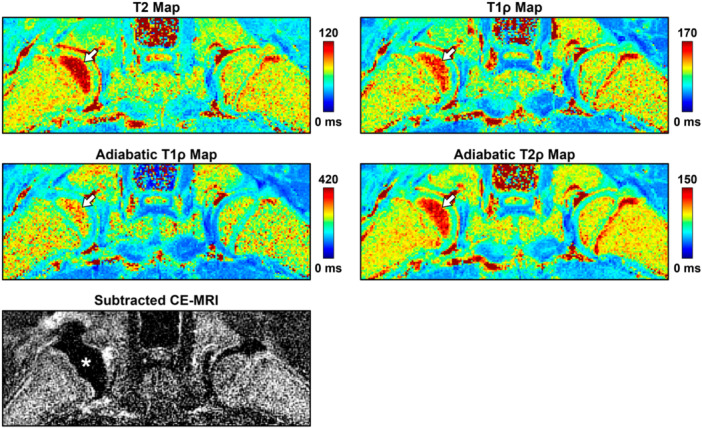
2D relaxation time maps and subtracted contrast‐enhanced MRI (CE‐MRI) image for a third piglet imaged one week after the onset of femoral head ischemia. The images are displayed as in Figures 5 and 6. This case had greatly increased T2, T1ρ, and adiabatic T2ρ relaxation times in both the peripheral and central subregions of the secondary ossification center. Adiabatic T1ρ was notably less sensitive to ischemic injury in this case.

2D relaxation time maps for three of the piglets are shown in Figures 5, 6, 7. In these representative cases, T2, T1ρ, aT2ρ, and (to a lesser degree) aT1ρ relaxation times were increased in the ischemic versus control SOCs. There was a more pronounced increase in relaxation times in the peripheral versus the central subregions of the SOCs.

## 4 Discussion

We found that T2, T1ρ, aT1ρ, and aT2ρ relaxation time mapping techniques are sensitive in detecting injury to the SOC of the femoral head in vivo 1 week after induction of ischemia in a piglet model of LCPD at clinical 3 T MRI field strength. T2 and aT2ρ relaxation times had the most pronounced increase in the ischemic versus contralateral‐control femoral heads, and aT2ρ relaxation times had the greatest effect size. Increases in T1ρ and (in particular) aT1ρ relaxation times in the ischemic SOC were more subtle than for T2 and aT2ρ. All four relaxation times had a greater increase in response to ischemic injury to the peripheral versus the central subregion of the SOC. These results support the potential utility of relaxation time mapping to evaluate the early, avascular stage of ONFH.

Our histological findings suggest that the observed increases in relaxation times were likely driven by bone marrow cell necrosis, including breakdown of cellular membranes and accompanying edema. This is consistent with an increase in molecular diffusion coefficients in the SOCs previously reported in these same piglets [32, 33], as well as relaxation time mapping studies of ischemic injury to brain, myocardium, and epiphyseal cartilage [30, 40, 41, 42, 43, 44].

A more pronounced increase in the relaxation times in the peripheral versus the central subregion of the injured SOCs was an unexpected finding. Plausible explanations for a greater susceptibility of the peripheral subregion to acute injury are its greater density of red marrow cells and vessels and greater metabolic demand associated with endochondral ossification. The peripheral subregion, which is likely specific to developing joints undergoing endochondral ossification, may be particularly noticeable in piglets on account of their rapid growth. The significance of our findings in the peripheral subregion of the SOC to the pathogenesis of LCPD and its relevance to children is a topic of interest for future study.

Our findings imply that T2 mapping may be more advantageous than T1ρ mapping in detecting early ischemic injury to bone marrow cells in vivo at clinical 3 T MRI field strength. Prior reports of T2 and T1ρ mapping in ex vivo femoral head specimens from LCPD piglet models imaged at 9.4 T field strength found that T1ρ relaxation times (using spin‐lock frequencies ranging from 250 to 1250 Hz, which includes the 350 Hz spin‐lock frequency used in the current study) were more prominently increased than T2 relaxation times in response to ischemic injury to the SOC [24, 25]. In the present study, we found the opposite: the increase in relaxation times was more pronounced for T2 than T1ρ. The reduced effect size of T1ρ in this study may be a result of differences in its sensitivity at 3 versus 9.4 T field strength. The sensitivity of T1ρ to factors affected by chemical exchange processes, such as acidosis resulting from necrosis, may be more pronounced at higher magnetic field strength [40, 45]. T1ρ is also less sensitive than T2 to the magnetic susceptibility of mineralized bone, the effect of which may be more pronounced at higher field strengths [46]. Further study is needed to determine the influence of these factors on relaxation times, including whether imaging at higher field strength may be a means to increase T1ρ sensitivity to ischemic injury to the SOC.

The consistency of aT2ρ mapping in detecting ischemic injury to the SOC observed in this study points to its potential utility as a robust measure of ischemic injury. While the percent increase in aT2ρ was similar to T2 in the SOC and its subregions, the standard deviation in relaxation time differences between the ischemic versus control femoral heads was nearly two‐fold lower for aT2ρ than T2, resulting in an almost two‐fold greater effect size. One possible explanation is that, compared to T2, aT2ρ has greater sensitivity to diffusion of water through local magnetic field gradients [28, 31], such as those caused by trabecular bone. This contrast mechanism could prove useful for evaluating changes to bone marrow in the presence of trabecular bone. Another possible explanation is that the use of adiabatic pulses for magnetization preparation provided a more robust means to measure aT2ρ than the Malcolm‐Levitt phase cycling approach used to measure T2, resulting in reduced variance in measurements between animals. In either case, our findings support that aT2ρ may be a particularly useful technique to evaluate ischemic injury to the SOC. Conversely, we found that aT1ρ mapping has relatively limited sensitivity in detecting early‐stage ischemic injury to the SOC. This can be attributed to the unique contrast mechanisms of this method, including its inherently longer relaxation times versus T2, continuous‐wave T1ρ, and aT2ρ [28, 29]. Additional investigations are needed to determine the contrast mechanisms responsible for the differences between the studied relaxation times and whether these differences may be useful to evaluate the SOC in later stages of LCPD or in other affected tissues of the femoral head.

Our findings support that T2 relaxation time mapping may be a clinically relevant technique to evaluate the early, avascular stage of ONFH. While a research magnetization‐prepared sequence was used in this work, T2 mapping using a multi‐echo spin echo sequence is widely available on commercial scanners, enabling T2 mapping to be applied in clinical settings. Compared to magnetization‐prepared sequences, a multi‐echo spin echo sequence can be acquired relatively quickly (albeit with limited through‐plane resolution), which may help make T2 mapping practical for inclusion in clinical workflows. T2 mapping may be particularly relevant to image pediatric patients with LCPD, given that, in addition to the SOC, it may also help inform pathological changes to the epiphyseal cartilage and metaphyseal spongiosa [26, 27]. In particular, T2 mapping may potentially serve as a complementary technique to CE‐MRI to inform the severity and extent of ischemic injury to the femoral head, independent of perfusion status. While we found that aT2ρ relaxation time mapping was more robust than T2 mapping in detecting ischemic injury to the SOC, this technique requires a slower magnetization‐prepared sequence, has higher radiofrequency heating than T2, and is not yet clinically available, which may limit its use to research settings in the near term.

Our study has two primary limitations that can be addressed in future work. First, the study was limited to imaging the piglet model at a single post‐operative time point, corresponding to avascular necrosis of red marrow cells. Patients with ONFH often present with chronic disease and partial involvement of the femoral head, which may result in unique changes that are not captured by our piglet model of acute and complete ischemia of the femoral head. A longitudinal study design with a longer post‐operative interval is needed to evaluate chronic effects of ischemic injury and subsequent reparative changes on relaxation times in the piglet model. Furthermore, children and adults may have substantial differences in bone marrow composition from the piglet model, including a greater degree of red‐to‐yellow marrow conversion. The results of our study are relevant to ischemic injury to red marrow cells, whereas the impact on adipocytes requires further investigation. Second, the contralateral femoral head was used as a control. While we do not expect marked changes to occur in the composition of the femoral head over the course of a single week, further study is needed to determine if changes in relaxation times were confounded by normal joint development. A longitudinal study design including pre‐operative imaging to track changes in the affected femoral head alone would address this deficiency.

In conclusion, T2, T1ρ, aT1ρ, and aT2ρ relaxation time mapping techniques are sensitive in detecting ischemic injury to bone marrow of the femoral head in vivo in a piglet model of LCPD at clinical 3 T MRI field strength. These relaxation time mapping techniques may provide a means to quantitatively evaluate the severity and monitor the progression of ischemic injury to bone marrow in pediatric and adult patients with ONFH. T2 mapping may be particularly useful as a clinically available quantitative MRI method, and aT2ρ mapping could provide additional advantage for robust imaging of ischemic injury. Changes in T1ρ and aT1ρ relaxation times were less pronounced, potentially limiting their utility for evaluating acute bone marrow necrosis at 3 T MRI.

## Author Contributions

Study design: Casey P. Johnson. Data acquisition: Casey P. Johnson, Erick O. Buko, Alexandra R. Armstrong, and Ferenc Tóth. Data analysis: Casey P. Johnson, Erick O. Buko, Sampada Bhave, Kayla L. Chase, Douglas Albrecht, and Alexandra R. Armstrong. Data interpretation: All authors. Manuscript drafting: Casey P. Johnson. Manuscript editing: All authors. All authors have read and approved the final submitted manuscript.

## Data Availability

The data that support the findings of this study are available from the corresponding author upon reasonable request.
